# The American Medical College Association—What It Is, and What It Should Be

**Published:** 1881-01

**Authors:** 


### Editorial.

### THE AMERICAN MEDICAL COLLEGE ASSOCIATION—WHAT IT IS, AND WHAT IT SHOULD BE.

In two preceding numbers of the JOURNAL AND EXAMINER, such facts have been adduced as show most conclusively that the suggestions put forth in the New York *Medical Record*, to the effect that the reason why some of the medical colleges in the "great Eastern cities" have not joined the American Medical College Association, and others have withdrawn from it, is because the Association is constituted chiefly of the "smaller colleges scattered throughout the West and South," and therefore could not adopt any important reforms without a fatal loss of membership, is entirely without foundation. On the contrary, the facts show that, in regard to every important reform in the system of medical education, the medical colleges "scattered throughout the West and South" have been ready and anxious to adopt all these reforms ever since the College Conventions of 1867 and 1870.

The facts also show, that the members of the present College Association in the West and South have advanced their lecture fees—have lengthened their College terms—have exacted the three years of medical study—have completely suppressed the practice that had existed in a few schools of giving two graduating courses of college instruction in each year; in a word, they have promptly complied with the Articles of Confederation adopted by the Association on the completion of its organization; and at the last meeting they took a most important step in advance, by so amending the Articles of Confederation that, after 1882–3, attendance upon *three* annual courses of college instruction should be required as a condition for final examination for the degree of M. D. Not one of the colleges in the great cities of Philadelphia, New York or Boston, have even proposed a measure of reform or advancement in a meeting of the Association since the completion of its organization in 1877 ; and, singularly enough, the only opposition made to the exaction of attendance on *three* annual courses of college instruction before graduation, by the Association, was inspired by the representatives of the only large medical school located in one of the great Eastern cities, that remained a member of the Association at the time the action was taken. With such a record of historical facts relating to the progress of events during the last fifteen years, the attempt to explain the conduct of the medical colleges in the "great Eastern cities" towards the American Medical College Association, on the pretense that such Association "can do them no good" on account of its inability to adopt any "important measure of reform without a fatal loss of membership," is something more than ludicrous. We have made the foregoing statements in defense of the College Association and the so-called "smaller colleges scattered throughout the West and South" that make up its present membership, solely in the interests of truth and justice. The Association is neither a child of our creation nor a special pet of our nursing. We joined it because its avowed object was to advance the cause of medical education ; and we have cordially sustained it because, thus far, it has steadily advanced in the direction of its avowed purpose, and not because we supposed it would do us or the school we represent any special good. If representatives of all the "larger schools in the great Eastern cities" had pursued the same course, thereby placing the medical colleges in all the larger cities of the country, East and West, North and South, in concert with each other, every important measure of reform or improvement in the system of medical education could have been carried into practical operation in less than five years, without the loss of any College members except such as are so located that they never should have been created. Inasmuch, however, as the large colleges in the great commercial centers of the Eastern part of the country have refused such co-operation, and thereby left the membership of the Association almost entirely in the West and South, let their friends and apologists hold them to their proper responsibility, and not make themselves ridiculous by attempting to shirk it upon others. That the refusal of the Eastern colleges to co-operate with, and share in, the direction and work of the Association is discouraging its friends and rendering its future progress uncertain, is apparent to all who bestow a thought upon the subject. That the withdrawal from membership of the New York and Philadelphia schools already mentioned will be followed by a withdrawal also of some schools in the West, we have no reason to doubt. Indeed, we are informed that the oldest medical college in this city, while enjoying the attendance of a class numbering four or five hundred, has already followed its Eastern examplers by notifying the Secretary of the Association of its withdrawal from membership. If the same course should be pursued by a few other schools in the West, it would certainly render the present organization of the Association powerless so far as the enforcement of just requirements are concerned, and yet an active and persistent maintenance of its organization and consistent advocacy of right measures might do much good in aiding to create and sustain a proper public sentiment, both in and out of the profession. In our estimation there is, and has been from the beginning, a radical defect in the organization of the Association.

As was intimated in our first editorial, in the October number, the Articles of Confederation establish a league or combination more for the purpose of correcting certain abuses than for the inaugurating of a reasonable standard of medical college education. These Articles, consequently, embody stringent rules against the issuing of scholarships and the various modes of pecuniary underbidding; against two graduating terms in one year; and for not less than three years of study, two annual courses of college instruction, and an examination in the seven principal branches of medical study.

But they give no definition of what should constitute a medical college, capable of imparting an adequate and proper medical education; neither do they indicate any standard of preliminary or general education that the student should comply with before commencing the study of medicine. These omissions of what should constitute the chief foundation of such an Association deprive it of all influence over the formation of new colleges, and of all power to maintain any standard of equality among its own members. Any six or eight doctors can club together and organize a college in a country village or at a road crossing, and though there may be neither a hospital or dispensary containing materials for clinical instruction, nor the slightest provision for practical instruction in chemistry, microscopy or physiology, yet if they issue a circular announcing that they observe the proper rules in regard to fees, require *three* years of study, two courses of lectures of not less than twenty weeks each, and an examination on the seven principal branches, they can at once apply for the admission of their institution to membership in the American Medical College Association, and there is nothing in the Constitution of the Association on which there can be founded any opposition to such admission. Experience has already shown that it is just this class of institutions that are most anxious to become members, because such recognition tends strongly to give them a degree of respectability they could not otherwise have. If, instead of standing aloof, the regular medical colleges in the larger cities of the East would directly unite with those similarly located in the chief cities of the West and South, in adopting such a standard of what should constitute the essentials of a medical college as is outlined in the following quotation from the report of a committee, made at the last meeting of the College Association, they would at once place our system of medical college education on a respectable basis, and would maintain a controlling influence over the character of all new college organizations. The part of the report to which we allude reads as follows:

"In considering what constitutes a proper standard for a college, we must determine: First, What constitutes a proper or adequate medical education? Second, What is the minimum length of time required for a fair acquisition of such an education? Third, What part of such time should be spent in direct attendance on a medical college? and, Fourth, What are the appliances, means of illustration, and facilities for imparting practical knowledge, that are necessary to constitute a college capable of performing all the functions required of such an institution.

"The first of these questions may be answered by saying that an adequate medical education is such as gives to the student a fair practical knowledge of all the branches of medical science, and a mental discipline sufficient for the proper use of such knowledge in the practice of the medical art. We use the words 'practical knowledge' as implying something more than mere verbal knowledge. For instance, a student might study a text-book on human anatomy until he could describe every bone in the skeleton, state the origin and insertion of every muscle, and give the distribution of the blood-vessels and nerves, and thereby sustain a fair verbal examination in that department. Yet, if he had never either dissected a subject himself, or witnessed the actual demonstration of each part by a competent teacher, he would be entirely devoid of that *practical* knowledge necessary to guide him in the operations of surgery. The same is true of most of the branches of medical science. Therefore, a practical knowledge of these various branches necessitates demonstrative teaching and personal manipulation, which can be provided in an adequate degree in medical colleges only. The amount of medical knowledge requiring for its proper acquisition this kind of teaching, makes it necessary that at least one-half of the whole time allotted as the period of medical pupilage should be spent in the medical college.

"As there are none at the present time who seriously claim that less than *three* full years should be devoted to diligent study of medicine, before graduation or commencement of practice, we may answer the second and third questions by stating that the minimum length of time required for gaining an adequate knowledge of medicine should be not less than three years; and that at least one-half of each of these years should be spent in a proper medical college. And this makes it necessary that the medical colleges, to be capable of performing their functions properly, should extend their annual term of active and obligatory instruction to six months of each year. It is obvious, also, that in answering the fourth question, we must insist that the very objects for which medical colleges are needed, make it necessary that each one should not only provide a full curriculum of studies, a full corps of teachers, and an annual term of six months' instruction, but also a sufficient supply of anatomical material for demonstrations and practical dissections; a chemical apparatus and laboratory for practical or analytical chemical work; a histological and physiological laboratory for practical and microscopic work; a museum containing a collection of specimens of normal and pathological anatomy sufficient for illustrating the departments of pathology, practical medicine and surgery; and access for the students to a hospital containing a daily average of not less than thirty patients actually in the wards for treatment and accessible for clinical instruction. A college which is so organized, or so located, that it does not or cannot provide for the daily use of its students all the resources here named, both for didactic and practical instruction, is not capable of performing all the functions that should be performed by every such institution that receives the patronage of the profession and the public. So long as the medical college diploma is recognized as a sufficient license to practice, every college asking for recognition should be required, in addition to possessing the foregoing means for adequate instruction in all departments, to exact proper evidence from every student that he possesses at least a good English education before allowing him to become a matriculate of the college; and after matriculation, he should be required to attend *three* regular annual terms of medical college instruction of not less than six months each, and the last of which should be in the institution granting him a diploma."

The adoption of such a standard as here indicated, by which the objects to be accomplished by a medical college and the means necessary for accomplishing them, should be clearly defined and adopted by the colleges in all parts of the country so located and organized as to be capable of doing so, would be promptly and permanently sustained by every important medical society organization; by the State Board of Examiners; and, so far as necessary, by State legislation. Will the colleges in the "great Eastern cities" make the necessary move in the matter, or will they continue to play the "dog in the manger," each for itself, and continue to make the so-called "smaller colleges scattered throughout the West and South," the *scape goats* for the sins that are pre-eminently their own? The responsibility is fairly upon *them*, and nothing can remove it but an open, manly co-operation on their part with such colleges in other parts of the country as have the necessary means for instruction, in adopting a proper standard of college organization, and maintaining it. D.

